# Development and Optimization of Bifunctional Fusion Proteins to Locally Modulate Complement Activation in Diseased Tissue

**DOI:** 10.3389/fimmu.2022.869725

**Published:** 2022-06-16

**Authors:** Kelly C. Fahnoe, Fei Liu, Jennifer G. Morgan, Sarah T. Ryan, Michael Storek, Ellen Garber Stark, Fred R. Taylor, V. Michael Holers, Joshua M. Thurman, Stefan Wawersik, Susan L. Kalled, Shelia M. Violette

**Affiliations:** ^1^ Preclinical Research Q32 Bio Inc., Waltham, MA, United States; ^2^ Division of Rheumatology, University of Colorado School of Medicine, Aurora, CO, United States; ^3^ Division of Renal Diseases and Hypertension, University of Colorado School of Medicine, Aurora, CO, United States

**Keywords:** anti-complement therapy, bifunctional fusion protein, complement negative regulatory protein, tissue targeting, C3d, pharmacodynamics, complement Receptor Type 1 (CR1), complement activation

## Abstract

Sustained complement activation is an underlying pathologic driver in many inflammatory and autoimmune diseases. Currently approved anti-complement therapies are directed at the systemic blockade of complement. Consequently, these therapies provide widespread inhibition of complement pathway activity, beyond the site of ongoing activation and the intended pharmacodynamic (PD) effects. Given the essential role for complement in both innate and adaptive immunity, there is a need for therapies that inhibit complement in diseased tissue while limiting systemic blockade. One potential approach focuses on the development of novel fusion proteins that enable tissue-targeted delivery of complement negative regulatory proteins. These therapies are expected to provide increased potency and prolonged tissue PD, decreased dosing frequency, and the potential for improved safety profiles. We created a library of bifunctional fusion proteins that direct a fragment of the complement negative regulator, complement receptor type 1 (CR1) to sites of tissue injury. Tissue targeting is accomplished through the binding of the fusion protein to complement C3 fragments that contain a surface-exposed C3d domain and which are covalently deposited on tissues where complement is being activated. To that end, we generated a fusion protein that contains an anti-C3d monoclonal antibody recombinantly linked to the first 10 consensus repeats of CR1 (CR1_1-10_) with the intention of delivering high local concentrations of this complement negative regulatory domain to tissue-bound complement C3 fragments iC3b, C3dg and C3d. Biochemical and *in vitro* characterization identified several fusion proteins that inhibit complement while maintaining the C3d domain binding properties of the parent monoclonal antibody. Preclinical *in vivo* studies further demonstrate that anti-C3d fusion proteins effectively distribute to injured tissue and reduce C3 fragment deposition for periods beyond 14 days. The *in vitro* and *in vivo* profiles support the further evaluation of C3d mAb-CR1_1-10_ as a novel approach to restore proper complement activation in diseased tissue in the absence of continuous systemic complement blockade.

## Introduction

While complement is an essential component of innate and adaptive immunity (1–3), uncontrolled and sustained complement activity plays a significant role in the pathogenesis of multiple human inflammatory and autoimmune diseases. Consequently, inhibition of complement has garnered significant attention as a therapeutic strategy for a wide range of diseases (1, 3–6).

**Figure 1:**
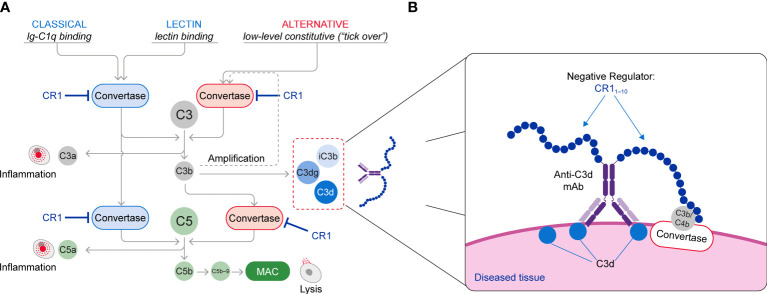
**(A)** Schematic of the Complement System (Classical, Lectin and Alternative Pathways) and **(B)** The Mechanism of Action of C3d mAb–CR1_1-10_. The C3d mAb portion of the fusion protein binds to C3d that is deposited on tissue at sites of complement activation and presents the negative regulator protein for binding to the C3/C5 convertases. Binding of the CR1_1-10_ negative regulator to the C3/C5 convertases induces their dissociation (decay accelerating activity) and catalytic degradation (co-factor activity).

The complement cascade is activated through three pathways: the classical pathway (CP) triggered by IgM and IgG immune complexes through C1 complex engagement; the lectin pathway (LP) whose engagement is mediated by binding of mannose-binding lectin to carbohydrate or glycoprotein components; and the alternative pathway (AP) that is spontaneously activated through the tick over mechanisms and also amplifies CP and LP complement activity **(** **Figure 1A** **)**. All three activation arms of the complement cascade drive formation of a C3 convertase complex, which cleaves the C3 protein into C3a and C3b. Formation of C3b increases complement activity both by triggering the amplification loop and by serving as an essential constituent of the C5 convertase complex, which catalyzes the late steps of complement activation common to all the complement pathways (7, 8). This terminal pathway activity leads to generation of C5a, which induces activation of pro-inflammatory pathways, and assembly of the membrane attack complex (MAC, C5b−9), which drives cell lysis (9, 10). Both C3a and C5a function as anaphylatoxins that recruit additional immune cells and further promote inflammation. C3b is inactivated and converted into ligands for complement receptors through cleavage by factor I, cofactors and proteases forming a series of fragments designated iC3b, C3dg and C3d (11).

Given the highly reactive nature of the complement system and its ability to quickly generate anaphylatoxins and damage cells, complement activity needs to be tightly regulated, especially on the cell surface, to avoid injury of host tissues (12). Complement regulatory proteins include complement receptor type 1 (CR1) (12), a naturally occurring complement negative regulatory protein that serves as a receptor for C3b, C4b, C1q, and recognition molecules of the LP (13, 14). CR1 can also bind to C3b and C4b and through both decay-acceleration and cofactor activity can functionally inactivate the CP, LP, and AP C3 and C5 convertases (2, 8, 15–17). Loss of regulation of either the convertase or the amplification loop leads to over-activation of the complement cascade. This, in turn, results in inflammation, tissue injury and impaired organ function that drives many chronic diseases (2, 8, 15–19).

Marketed and clinical stage anti-complement therapies predominantly target systemic blockade of complement activity. Given complement’s essential role in the innate immune system, however, such therapies are generally restricted to rare, life-threatening diseases. A further challenge is the fact that components of the complement cascade exist in high abundance and undergo rapid systemic turnover, making effective inhibition difficult. Consequently, there is a substantial unmet need for safer and more effective anti-complement therapies. One potential strategy to bypass systemic complement blockade and provide potent therapeutic benefit involves targeting the complement inhibitor to diseased tissue, where complement is overactivated. A similar approach using fusion proteins containing a fragment of human complement receptor type 2 (CR2) and either the first 5 consensus repeats of human factor H (fH_1-5_) or the first 10 consensus repeats of CR1 (CR1_1-10_), has been described previously (20, 21). These molecules progressed from preclinical studies to a phase 1 study in patients with paroxysmal nocturnal hemoglobinuria (PNH), but their clinical development appears to have been limited by pharmacokinetic (PK) and pharmacodynamic (PD) properties (20, 22). Nevertheless, these molecules provide an important basis for a tissue targeted approach that relies on binding to C3 complement fragments resident in tissues at sites of complement activation.

The approach described here focuses on generating monoclonal antibody (mAb)-derived bifunctional recombinant fusion proteins, engineered to contain two moieties of CR1_1-10_ linked *via* a non-antigenic (Gly4Ser)_2_ sequence to an anti-C3d mAb. CR1_1-10_ has been previously shown to bind to both CP and AP convertases and potently lead to their inactivation through decay accelerating activity and cofactor activity (23–25). These fusion proteins were designed to target and deliver high local concentrations of CR1_1-10_ to diseased tissue at sites where complement is activated. The C3d antibody portion was generated from mice with a targeted deletion of the *C3* gene (C3*^-/-^* mice) immunized with recombinant human C3d (26). The selected clone, 3d8b, binds with high affinity to a common epitope on complement fragments iC3b, C3dg and C3d, which are deposited at high density at sites of complement activation (27–30). We subsequently use the term “anti-C3d” when describing the binding activity of the antibody and targeted fusion proteins.

This approach brings the CR1_1-10_ regulatory protein into proximity with surface-bound C3/C5 convertases, resulting in potent inactivation of these effector complexes and interruption of further complement activation **(** **Figure 1B** **)**. Consequently, control of the complement system at specific sites of ongoing injury is restored without systemic blockade of complement activity.

We describe in detail the generation and characterization of the C3d mAb–CR1_1-10_ fusion proteins. In addition, we demonstrate the therapeutic potential of these molecules by providing data from *in vivo* studies that investigate tissue specificity and PK/PD properties. Given that injured and diseased tissues where complement is activated are characterized by deposition of C3d, the use of these targeted fusion proteins provides a treatment strategy for multiple clinical indications. Thus, this therapeutic approach can provide tissue selective complement inhibition while minimizing circulating drug exposure that may affect immune surveillance or other homeostatic functions mediated by systemic complement activity.

## Materials and Methods

### Design, Expression and Purification of Antibody Fusion Proteins and Controls

Anti-C3d mAb, clone 3d8b, was originally identified as a murine IgG2b mAb (26). For initial *in vitro* and *in vivo* assays, we expressed the 3d8b mAb as a mouse IgG1. We then constructed a panel of C3d mAb-CR1_1-10_ fusions in different configurations. The CR1_1-10_ domain contains the first 10 consensus repeats of the human CR1 protein (P17927, www.unitprot.org) and was recombinantly expressed on the N- or C-terminal ends of the C3d mAb heavy and light chains with a (Gly4Ser)_2_ linker located between the antibody and the CR_1-10_ domain. Fusion proteins that contain one negative regulatory protein are referred to as a heterodimer and fusion proteins containing two regulatory proteins are referred to as a homodimer. Heavy and light chain constructs were transfected and transiently expressed in Chinese hamster ovary (CHO) cells. Expression of a heterodimer fusion protein required the transfection of three constructs (the heavy chain with and without the CR1_1-10_ domain and a common light chain) and were designed using knob-into-hole substitutions in the Fc domain to drive the formation of the heterodimeric construct (31). Whereas expression of the homodimer fusion proteins only required the co-transfection of one heavy and one light chain fusion construct, resulting in only one possibility for heavy and light chain pairing. For studies with the humanized C3d mAb, the complementarity-determining regions (CDRs) of 3d8b were grafted onto a hinge stabilized (S228P), Fc-mutated (L235E) human IgG4 isotype, selected due to its low effector function and negligible potential for C1q binding and subsequent complement activation. Following gene synthesis, anti-C3d parent antibodies, antibody fusions, Fc-fusions and soluble proteins were transiently expressed in CHO cells using standard culture methods. Clarified supernatants were affinity purified using Protein A or immobilized metal ion affinity chromatography (IMAC; Cytiva, Marlborough, MA) and buffer exchanged into phosphate-buffered saline (PBS) using size exclusion chromatography to yield material with greater than 95% purity (**Supplementary Figure 1**) and endotoxin levels below 0.5 EU/mg.

### Linear Peptide Mapping

Using PEPperCHIP^®^ custom peptide microarrays (PEPperPRINT, Heidelberg, Germany), the full-length sequence of human C3d was evaluated with GSGSGSGS added at the N- and C- terminus, to avoid truncated peptides. This sequence was translated into 15 amino acid linear peptides with a peptide–peptide overlap of 14 amino acids. The C3d peptide microarrays were printed synthetically as duplicate linear microarrays. Hemagglutinin (HA) control peptides, plus 15 unrelated peptides were included to monitor assay quality and microarray integrity. In addition, isotype control and anti-C4d antibodies were included as negative controls. After blocking, the microarrays were incubated with 1, 10 and 100 µg/mL of anti-C3d mouse antibody followed by staining with DyLight™ goat anti-mouse secondary and mouse anti-HA control antibodies (Thermo Fisher Scientific, Waltham, MA). All incubation steps were performed with reagents diluted in PBS, pH 7.4 + 0.05% Tween^®^ 20 (PBS-T) plus 10% blocking buffer (MB-070, Rockland Immunochemicals Inc., Philadelphia, PA). After thorough washing, the microarrays were imaged using near-infrared imaging (LI-COR Odyssey Imaging Systems, Lincoln, NE). Microarray image analysis was performed using the PepSlide^®^ Analyzer to quantify spot intensities and annotate peptides. An intensity map of averaged intensities from duplicates corrected for fore and background signals was generated.

### Crystallization of Fab (3d8b) With Human C3d

Crystal studies of the Fab(3d8b):C3d complex (XTAL BioStructures, Inc., a fully owned subsidiary of Schrödinger, Inc., Natick, MA) were performed using sparse matrix screening of commercially available reagent kits. Sitting-drop vapor diffusion experiments were set up with equivalent volumes of 3d8b antigen-binding Fragment (Fab) and reservoir solution in each drop and incubated at 20°C. Multiple needle-shaped hits were identified after 48 hours in conditions with combinations of polyethylene glycol (PEG) of various lengths and buffers in a pH range of 7.0 to 8.5, with no additional salt component. Based on primary crystal thickness, select conditions were optimized. Rod-like crystals were harvested in microfiber loops and drawn through cryoprotectant. Harvested crystals were flash-cooled in liquid nitrogen and x-ray diffraction data were measured at beamline BL13-XALOC (ALBA synchrotron, Barcelona, Spain) at a single wavelength of 0.979257 Å using a Dectris Pilatus 6M detector (Dectris, Baden-Daettwil, Switzerland). The structure was determined from the crystal with the highest resolution (1.75 Å) and data quality using molecular replacement models from published structures of an IgG4 derived Fab fragment (PDB ID: 5DK3) and C3d (PDB ID: 4ONT), stripped of all ligands and solvent molecules. Additional model building and refinement was conducted in Coot and Refmac, respectively. Final refinement statistics are shown in **Table 1**.

**Table 1:** Model refinement statistics for Fab(3d8b):C3d complex (PDB ID 7UE9).

Parameter	Description	
Refinement range (Å)	49.47−1.75	
R_cryst_ (%)	16.92	
R_free_ (%)	20.16	
Ligands (per asymmetric unit)		
Fab(3d8b)	1	
C3d	1	
Bond lengths, rmsd (Å)	0.015	
Bond angles, rmsd (°)	1.995	
Average B-factors (Å^2^)	Main chain atoms	Side chain atoms
Heavy chain (H)	15.15	17.43
Light chain (L)	18.10	21.60
C3d (C)	19.89	24.10
Ramachandran plot (%)		
Preferred	97.78	
Allowed	1.81	
Outliers	0.42	

Rmsd, Root mean square deviation.

### C3d Binding Assays

#### Enzyme-Linked Immunosorbent Assay

Protein binding to C3d was evaluated by indirect enzyme-linked immunosorbent assay (ELISA). Human C3d protein (Complement Technologies, Tyler, TX) was coated on the surface of 96-well plates (26) overnight at 4°C, then washed in PBS-T and blocked in PBS plus 2% bovine serum albumin (BSA) at ambient temperature for ≥2 hours or overnight at 4°C. Samples containing C3d-binding proteins diluted in PBS plus 0.1% BSA were added, and plates were incubated at ambient temperature for 1 hour, washed in PBS-T, and then probed with horseradish peroxidase (HRP) -conjugated secondary antibody. The specific secondary antibody used was dependent on the binding protein isotype and design (rabbit anti-mouse kappa light chain, mouse anti-human pFc’, or anti-CR1-biotin/streptavidin HRP). After thorough washing in PBS-T the binding of the peroxidase-conjugated secondary antibody was quantified by the addition of a developing solution, 3,3’,5,5’-tetramethyl-benzidine (TMB) for 10–15 minutes. To stabilize the signal a stop solution (maleic acid) was added to amplify the signal and convert to a colorimetric readout at 450 nm. Mouse C3d was substituted for human C3d to test species cross-reactivity under the same conditions. Half maximal effective concentration (EC_50_) curves were generated using four-parameter (variable slope) non-linear regression in GraphPad PRISM v9.0 (GraphPad Software, San Diego, CA).

#### Surface Plasmon Resonance

Binding affinity measurements to C3d were performed by Surface Plasmon Resonance (SPR) on Biacore 3000 at 25°C. A CM5 (carboxymethylated dextran) chip surface was equilibrated as per manufacturer protocol (Cytiva, Marlborough, MA) in 10 mM HEPES, 150 mM NaCl, 3 mM EDTA and 0.05% v/v Surfactant P20 (HBS-EP+). Flow cell 2, 3 and 4 of the CM5 chip were coated with low surface density (20-30 Resonance Units, RU) of human C3d using a standard EDC/NHS amine coupling method in 10 mM sodium acetate pH 5.0. Flow cell 1 was activated and left blank as a reference channel. 1M ethanolamine was injected over each flow cell to block any unoccupied binding sites. Anti-C3d antibodies and fusion proteins were diluted in HBS-EP+ running buffer in a concentration series ranging between 0-200 nM and injected over surface bound target antigen at a flow rate of 30 µL/min for 120 s followed by dissociation in running buffer for 180 s. Binding of antibody fusion proteins was monitored in real time and fit using Langmuir (1:1) binding model. From the observed *k*_on_ and *k*_off_, K_D_ was determined. Surfaces were regenerated using two injections of 10mM glycine at pH 1.7 for 40 s.

### Functional Activity

#### Complement Activation Assays

Complement activation assays were performed using the Wieslab^®^ AP and CP ELISAs (SVAR Life Science AB, Malmö, Sweden) according to manufacturer’s instructions. Briefly, complement preserved human serum was diluted 1:18 or 1:101 in the appropriate dilution buffer provided. Antibody fusion proteins were prepared at 25-fold their final concentrations in PBS pH 7.4 and serially diluted two- to four-fold for an eight-point dose response, then added to the diluted human serum at their final concentrations and transferred to pre-coated ELISA plates. The plates were incubated for 1 hour at 37°C and washed three times. Alkaline phosphatase conjugated anti-C5b−9 secondary antibody was added, and the plates incubated at ambient temperature for 30 minutes, then washed three times. Following addition of a colorimetric substrate, plates were incubated in the dark at ambient temperature for a further 30 minutes before absorbance was read at 405 nm. Half maximal inhibitory concentration (IC_50_) curves were generated using four-parameter (variable slope) non-linear regression in GraphPad PRISM 9.0 and extrapolated for 100% serum.

#### Alternative Pathway Hemolysis (AH50) Assay

Rabbit red blood cells (RRBCs) were washed in PBS without Ca^2+^/Mg^2+^ and resuspended to 2.9 x 10^9^ cells/mL. Antibody fusion proteins were serially diluted in complement preserved normal human serum (100 µL) to concentrations between 0.0195 µM and 10 µM. Mg^2+^-EGTA buffer (10 µL) was then added to the serum/antibody mixture to direct complement activation through the AP, followed by addition of 1.5 x 10^7^ prepared RRBC’s (5 µL). The reaction was incubated for 30 minutes at 37°C. Reactions were stopped with the addition of 25 mM ethylenediaminetetraacetic acid (EDTA; 115 µL) and plates were centrifuged (1800 rpm) to separate lysed cells. Supernatant (100 µL) was transferred to a 96-well clear-bottom plate and absorbance at 415 nm measured. Complement inhibition was assessed as decreased RRBC lysis versus untreated controls. AH50 curves were generated using GraphPad PRISM v9.0 by subtracting the background of serum only wells and normalizing to lysis and untreated control values.

#### Classical Pathway Hemolysis (CH50) Assay

Sheep red blood cells (SRBCs) were washed in gelatin veronal buffer with MgCl_2_/CaCl_2_ (GVB^++^) and isolated by centrifugation. Pelleted SRBCs were washed and diluted two-fold in GVB^++^. Resuspended cells were sensitized with hemolysin (rabbit anti-sheep erythrocyte antiserum) for 30 minutes at 30°C (32). CH50 assays were conducted using serial dilutions of normal human serum in GVB^++^ to determine the amount of serum required to lyse sensitized SRBCs (sSRBCs). To measure hemolysis, 200 µL of sSRBCs were incubated for 30 minutes at 37°C with 100 µL GVB^++^ and 100 µL of diluted complement-preserved human serum (concentration optimized during the CH50 assay) to direct complement activation through the CP. In these studies, antibody fusion proteins were serially diluted to concentrations between 195 pM and 1 µM in complement preserved normal human serum before addition of sSRBC and GVB^++^. Assay plates were centrifuged (2000 rpm) to separate lysed cells, and 100 µL of supernatant was transferred to a 96-well clear bottom plate containing 100 µL of water and absorbance at 540 nm was recorded. Complement inhibition was assessed as decreased sSRBC lysis versus untreated controls. CH50 curves were generated using GraphPad PRISM 9.0 by subtracting the background of serum only wells and normalizing to values of untreated controls where full lysis resulted and extrapolated for 100% serum.

#### Zymosan Complement Activation Assay

Pre-activated zymosan (Complement Technologies, Tyler, TX) was diluted eight-fold in PBS, pH 7.4 containing 25 mM EGTA, 12.5 mM MgCl_2_ and 0.1% BSA final concentrations. Antibody fusion proteins were prepared at five-fold their starting concentration and serially diluted in PBS pH 7.4, 0.1% BSA. To initiate the AP, 40 µL of activated zymosan diluted in Mg^2+^-EGTA-containing buffer was combined with 20 µL of fusion protein and 40 µL of complement preserved serum diluted to 25% in PBS pH 7.4, 0.1% BSA. Assay controls replaced the fusion protein with equal volumes of 50 mM EDTA (negative) and PBS, pH 7.4, 0.1% BSA (positive). Reactions were incubated at 37°C for 20 minutes and stopped by the addition of 20 µL of 50 mM EDTA. Assay supernatant was removed by centrifugation (3000 rpm), and the zymosan pellet was resuspended and washed three times with PBS pH 7.4, 0.1% BSA followed by centrifugation. The final pellet was resuspended in 100 µL goat anti-mouse C3−fluorescein isothiocyanate (FITC) F(ab’)2 (MP Biomedicals, Solon, OH) prepared in PBS, pH 7.4 and incubated for 1 hour on ice. Following washing as described above, the pellets were resuspended in 200 µL PBS, pH 7.4, 0.1% BSA. Data were acquired on an Attune flow cytometer (ThermoFisher, Waltham, MA) collecting 10,000 events per well in autosampler mode, and were analyzed in FlowJo (FlowJo, LLC, Ashland, OR). Percentage inhibition was calculated based on controls, while half maximal inhibitory concentration (IC50) curves were generated using four-parameter (variable slope) non-linear regression in GraphPad PRISM v9.0 and extrapolated for 100% serum.

#### Cell Injury Assay

Human microvascular endothelial cells (HMEC, ATCC CRL-3243) were seeded on collagen-coated 96-well plates at approximately 64,000 cells per well in 200 µL of complete culture media (MCDB-131 basal media supplemented with 10% heat-inactivated fetal bovine serum [FBS], 1 µg/mL hydrocortisone, 1 U/mL penicillin/streptomycin + L-glutamine, 50 µg/mL endothelial cell growth factor [ECGF]). Cells were cultured to confluency for 48−72 hours. Cells were gently washed with test media (Hank’s balanced salt solution, 0.1% dextrose, 28 mM Trizma base, 0.5% BSA, pH 7.3) three times before incubation for 10 minutes at 37°C/5% CO_2_ in 50 µL of 100 mM H_2_O_2_ (or 10 µM adenosine diphosphate [ADP]) to induce injury. Cells were immediately washed with test media and incubated with 25% complement preserved human serum with or without a dose titration of C3d mAb-fusion proteins, soluble CR1_1-10_ or C3d mAb. Test proteins were incubated on the injured cells for 60 minutes at 37°C/5% CO_2_, and the cells were then washed with test media three times. Cells were fixed in 4% paraformaldehyde diluted in test media for 10 minutes at ambient temperature, followed by three washes in test media before blocking for 30 minutes at ambient temperature with 5% BSA diluted in test media. The cells were washed once more with test media before incubating with FITC-rabbit anti-human C3c (Dako, Santa Clara, CA) for 60 minutes at ambient temperature shielded from light. Following three washes in test media, the cells were counterstained with DAPI diluted in PBS for 5 minutes in the dark. The DAPI stain was removed and replaced with PBS prior to imaging on Cytation 1 cell imaging multi-mode reader (BioTek, Winooski, VT) at 10x magnification in the FITC channel. Fluorescence images were processed and analyzed with Gen5 software using total fluorescence normalized to total number of cells per well.

### Animal Models

#### C57BL/6 Mice

C57BL/6 mice for PK studies were housed and cared for by Biomere (Worcester, MA) according to Internal Standard Operating Procedures under guidance by Biomere IACUC and in accordance with Association for Assessment and Accreditation of Laboratory Animal Care standards. Male and female mice aged >8 weeks were used on study.

#### fH Deficient (*CfH* ^-/-^) Mice

C57BL/6 fH-deficient (*CfH* ^-/-^) mice carrying a targeted disruption of the gene encoding fH were generously provided by Prof. Matthew Pickering (Imperial College, London, UK) (33, 34). Loss of functional fH protein results in increased complement activation and C3d deposition in the kidneys and spontaneous development of complement-mediated renal injury (33, 34). All mice were housed and cared for by Biomere according to Internal Standard Operating Procedures under guidance by Biomere IACUC and in accordance with Association for Assessment and Accreditation of Laboratory Animal Care standards. Male and female mice aged 10−45 weeks were used for PK/PD and efficacy studies.

### Mouse Circulating PK

To investigate circulating PK of the fusion protein using *in vivo* mouse models, we generated a mouse surrogate of C3d mAb–CR1_1-10_ that contains the same anti-C3d mAb, 3d8b, linked to Crry, the murine complement CP/AP/LP inhibitory molecule that is the functional orthologue of human CR1 in its ability to perform decay-acceleration and cofactor activity (35). Two moieties of Crry were linked to the C-termini of the heavy chains of 3d8b with (Gly_4_Ser)_2_ peptide linkers to generate a C3d mAb–Crry fusion protein. C3d mAb−Crry was administered to C57BL/6 mice (two males/two females) by intravenous (IV) injection at 50 mg/kg and blood samples were collected pre- and 2, 6, 24, 30, 48, 72, 144 and 168 hours post-dose. Blood was collected into pre-chilled K_2_EDTA tubes. Tubes remained on ice for up to 15 minutes after collection until centrifugation at 2000 rpm, at 4°C. Plasma was collected and immediately snap frozen in liquid nitrogen and stored at -70°C.

Plasma samples were analyzed for the concentration of C3d mAb−Crry using a qualified ELISA-based analytical procedure. Plates were coated with 100 µL/well of C3d at a concentration of 2.5 µg/mL in BupH carbonate–bicarbonate buffer (Thermo Scientific, Waltham, MA), washed with Tris-buffered saline plus 0.05% Tween 20 (TBST) (Boston BioProducts, Ashland, MA) and then blocked with TBST with 1% BSA. Block was removed and 100 µL of TBST-diluted mouse plasma or C3d mAb−Crry standards diluted into TBST/plasma were added to wells and incubated for 2 hours. Plates were washed with TBST and then incubated with rat anti-mouse Crry (Hycult Biotech, Uden, The Netherlands) diluted in TBST + 1% BSA, washed and then incubated with HRP-conjugated AffiniPure donkey anti-rat IgG (H + L) secondary detection reagent (Jackson ImmunoResearch, West Grove, PA), followed by detection with TMB chromogenic solution. Following addition of stop solution (R&D systems, Minneapolis, MN), absorbance was measured at 450 nm. PK parameters were derived using non-compartmental analysis (NCA) methods within Phoenix WinNonLin^®^ version 8.0 (Certara USA, Inc., Princeton, NJ). The non-compartmental PK parameters reported were maximum plasma concentration (C_max_), time to maximum plasma concentration (T_max_), area under the curve from time zero to the last measurable concentration (AUC_0–last_), AUC from time zero extrapolated to infinity (AUC_0–inf_), mean residence time (MRT), half-life (t_1/2_), goodness-of-fit statistic for the terminal elimination phase (Rsq), clearance (CL), elimination rate constant (λ_Z_), elimination volume of distribution (V_z_), steady state volume of distribution (V_ss_).

### Tissue PK/PD

#### Target Engagement in *CfH^-/-^* Mice

To assess tissue target engagement in *CfH* ^-/-^ mice over a period >10 days and avoid immunogenicity, the mouse surrogate fusion protein, C3d mAb−Crry (60 mg/kg), and a molar matched non-C3d targeted control, mFc−Crry (34.2 mg/kg), were administered on day 1 and day 2 by IV tail vein injection and mice were euthanized at the specified timepoints. Blood was collected pre-dose and at study end and processed for plasma and serum. The right kidneys were harvested at 14 days post-second dose and frozen in optimal cutting temperature (OCT) compound for immunostaining. Kidney tissue sections were incubated with primary antibody at ambient temperature for 30 minutes. The sections were washed three times with Dulbecco’s PBS (DPBS) before incubation with a secondary antibody for 30 minutes, and then washed a further three times with DPBS and mounted with permanent mounting media. To confirm that C3d mAb alone did not have pharmacologic effects or inhibit detection of complement activation, 3d8b was administered at a molar matched dose (16.9 mg/kg) on days 1 and 2 by subcutaneous injection. Mice were euthanized and kidneys harvested 7 days post dose and prepared as described above. An additional study using molar matched doses of humanized C3d-mAb–CR1_1-10_ (10 mg/kg) and humanized C3d mAb (5 mg/kg) was conducted at a single intravenous dose and kidneys were harvested 7 days post-dosing using identical kidney preparations as above and immunostained to evaluate target engagement and complement activation.

Fluorescent immunostaining with a Crry-specific antibody (anti-mouse Crry/p65, clone 8A/E6) or an anti-human IgG4 antibody (mouse anti-human IgG4 pFc’, clone HP6023) was used to detect target engagement, while complement activity was measured using an antibody specific for active C3 cleavage products (MP Biomedicals, Solon, OH). Immunofluorescence images were acquired at 40x magnification with the EVOS FL cell imaging system (Life Technologies, USA) and quantified using ImageJ v1.53n to measure mean fluorescence intensity from a minimum of 6 glomeruli per animal (3−4 animals per time point).

## Results

### Epitope Mapping

C3d targeting is achieved using an anti-C3d mouse monoclonal antibody clone, 3d8b, which was generated by immunizing C3-deficient mice with human C3d protein and then selected for binding to mouse and human C3d, iC3b and C3dg without binding to intact C3 or C3b (26).

To refine our understanding of the epitope to which the3d8b mAb binds,we carried out two orthogonal epitopemapping studies, linear peptide screening and cocrystallization. Peptide screening showed very strong 3d8b binding with high spot intensities and signal-to-noise ratios against adjacent peptides with the consensus motif NLDVSLQLPS. This peptide sequence is located at the C-terminus of C3d and is contained within the related protein fragments iC3b and C3dg. 3d8b showed no binding signal to any of the 15 negative control peptides. The negative control antibodies, mouse IgG1 isotype and anti-C4d, showed no binding signal to any of the C3d peptides.

**Figure 2:**
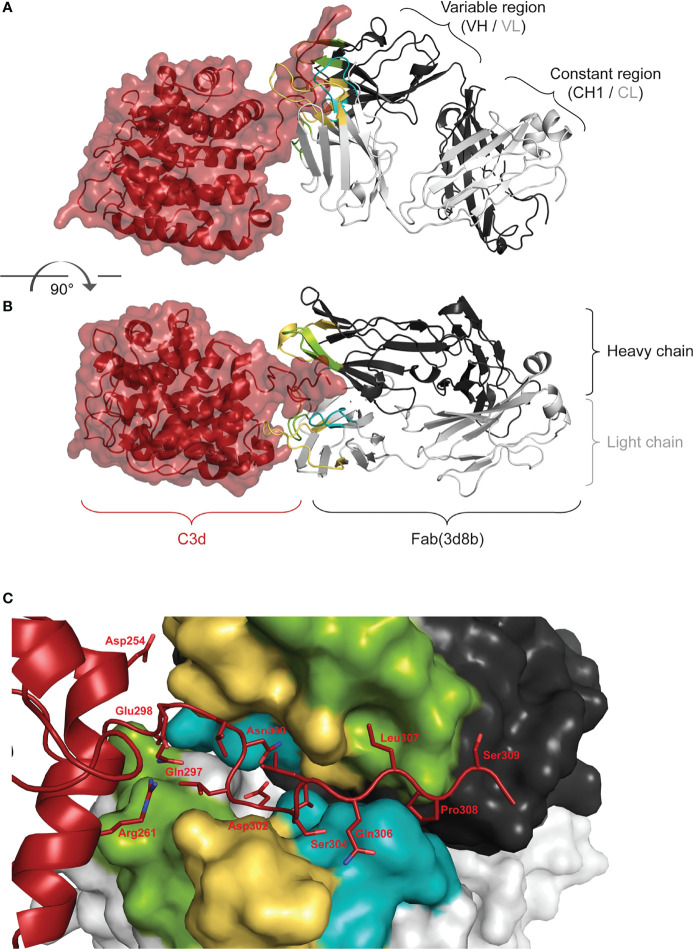
Fab (3d8b) in Complex with human C3d. *Paratope* Fab heavy chain is shown in dark gray and the light chain in light gray, with CDRs H1/L1 in yellow, H2/L2 in green, and H3/L3 in teal; C3d is shown in dark red. Panel **(A)** is rotated approximately 90° to generate the view in panel **(B)** *Epitope* Panel **(C)** illustrates the extension of the C-terminal of C3d into the cleft between the heavy and light chain of the Fab (3d8b). Fab (3d8b) surfaces are shown for the heavy chain (dark gray) and the light chain (light gray), with CDRs highlighted in yellow (H1 and L1), green (H2 and L2), and cyan (H3 and L3). C3d α-carbon backbone is shown in dark red with side chain sticks (oxygen - red, nitrogen - blue) for residues within 5 Å of the Fab.

Co-crystallization studies of the humanized Fab(3d8b) with C3d were performed in parallel with peptide mapping. Crystallization studies can better distinguish conformational epitopes and allow the antibody paratope to be determined. Data collected from high resolution crystals (PDB 7UE9) showed that Fab(3d8b) binds C3d almost exclusively at the C-terminal extended chain (amino acids [aa] 295−309), a section of C3d that extends outside of the globular fold formed by the bulk of the protein (aa 1−294) **(** **Figures 2A, B** **)**. This C-terminal extended chain binds along the cleft between the heavy and light chains at the variable region **(** **Figure 2C** **)**. The C3d:Fab(3d8b) interaction is accomplished through an extensive binding surface that offers shape and charge complementarity to the C-terminal C3d epitope. This involves residues from all six Fab CDRs as well as a key salt bridge between a framework residue, Arg51 (Light Chain), and C3d Asp302. The C3d epitope is locked in place by a salt bridge, ten H-bonds, and numerous water-mediated H-bonds and Van der Waals interactions.

There are two Fab contacts at the globular portion of C3d, outside of the C-terminal epitope described above. However, the impact of these two contacts appear to be negligible in their contribution to the overall binding of the Fab to C3d. Thus, the main interactions between C3d and the Fab are formed by C3d C-terminal residues 295 to 310, consisting of contiguous or near contiguous residues DHQELNLDVSLQLPS. A similar sequence with 93% and 67% identity is found in cynomolgus and mouse C3d, respectively.

These orthogonal data confirmed that the main interactions between C3d and the 3d8b Fab are formed by C3d C-terminal residues 295 to 310 and this epitope is considered linear, comprising contiguous or near contiguous residues. This included NLDVSLQLPS identified by linear peptide mapping and DHQELNLDVSLQLPS identified by the co-crystal structure. Further information gathered from the co-crystal structural study identified the paratope, showing engagement of all six CDRs.

### C3d mAb and Fusion Protein Binding to C3d

**Figure 3:**
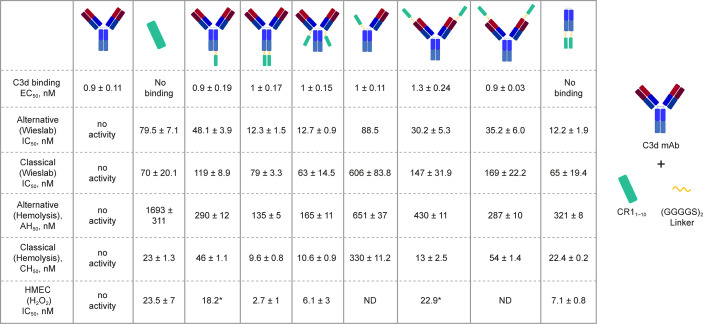
C3d binding and complement inhibitory activity of test proteins. Binding affinity to C3d was evaluated by ELISA. *In vitro* functional potency of proteins was assessed using three complement activity assays: the Wieslab AP and CP complement assays, the AP and CP red blood cell (RBC) lysis assays, and the HMEC assay with H_2_O_2_ induced injury. Schematics at the top of each data column indicate the orientation of the CR1_1-10_ effector molecule (cyan rectangle). Where only a single dataset was collected, the value is denoted with “*”.

We generated a panel of recombinant bifunctional fusion proteins (C3d mAb–CR1_1-10_) containing anti-C3d mAb 3d8b linked via a non-immunogenic flexible (Gly_4_Ser)_2 _peptide to CR1_1-10_ in a variety of configurations (**Figure 3**). This evaluation included linking CR1_1-10_ to the N- or C-terminus of one or both heavy chains, or to both light chains of the C3d mAb. These prototype fusion proteins were designed to evaluate whether the positioning of CR1_1-10_ on the C3d mAb affected binding to the C3d target or the activity of the CR1_1-10_ complement negative regulatory protein. The binding affinity of the C3d mAb–CR1_1-10_ fusion proteins to C3d were compared to that of 3d8b alone and non-targeted controls including soluble CR1_1-10_ (sCR1_1-10_) and Fc-CR1_1-10_. Proteins were evaluated for binding to C3d by ELISA.

C3d mAb−fusion proteins containing CR1_1-10_ linked to the N- or C-termini of 3d8b heavy or light chain retained similar C3d binding affinity as compared to 3d8b alone (**Figure 3**). As expected, sCR1_1-10_ and Fc−CR1_1-10_, showed no detectable binding to C3d. To further understand the true binding affinity (K_D_) to C3d for the lead construct designs, C3d mAb–CR1_1-10_ and C3d mAb-Crry, and for C3d mAb alone, we performed SPR studies (**Supplementary Figure 2**). By SPR analysis, the three different proteins demonstrated K_D_ values ranging from 10 to 27 nM (as also shown in **Table 2**). Consistent with the ELISA results, there was no major difference in the C3d binding affinity for the two fusion proteins as compared to C3d mAb alone.

**Table 2:** Binding affinity to C3d and functional activity of C3d mAb, Fc-Crry, C3d mAb–Crry and C3d mAb – CR1_1-10_.

Assay	C3d mAb	Fc−Crry	C3d mAb−Crry	C3d mAb−CR1_1-10_
C3d binding ELISA EC_50_, nM	0.9	NB	0.7	1
C3d Binding SPR K_D_, nM	12.9	NB	10.6	27.8
Zymosan Alternative Pathway IC_50_, nM*	NA	330	140	52

Binding affinity to C3d was measured by both Biacore and ELISA. C3d mAb−Crry maintains similar binding affinity to C3d as the parent C3d mAb, 3d8b. Fc−Crry, C3d mAb–Crry and C3d mAb-CR1_1-10_ were evaluated for the ability to inhibit complement activity in a mouse serum zymosan assay.

EC_50_, half maximal effective concentration; IC_50_, half maximal inhibitory concentration; K_D_, equilibrium dissociation constant; NA, no activity; mAb, monoclonal antibody; NB, no binding; SPR, surface plasmon resonance.

*Zymosan assay is conducted in 10% mouse serum, extrapolation to 100% serum is achieved by multiplying the IC_50_ value by 10.

### *In Vitro* Complement Inhibitory Activity

**Figure 4:**
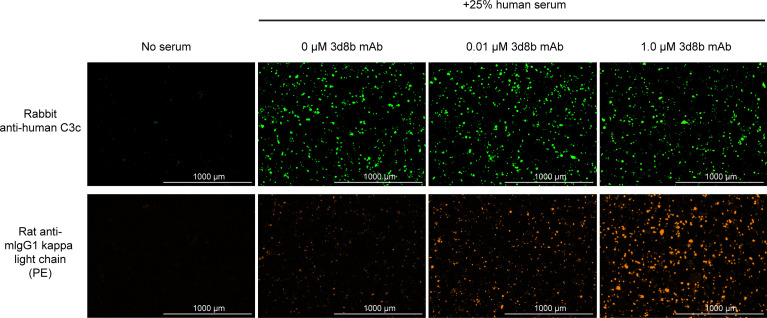
Complement activation and C3d deposition in the HMEC assay. Anti-C3 fragment (green) and anti-3d8b mAb (red) immunostaining of injured HMEC cells with human serum complement activation. Immunostaining is shown with or without human serum treatment and with or without co-culture of anti-C3d mAb, 3d8b, at the indicated concentrations.

*In vitro* complement inhibition by C3d mAb−CR1_1-10_ fusion proteins was assessed using three complement activity assays: the Wieslab assay, which detects the formation of terminal complement complex, C5b−9, induced with human serum; the hemolysis assay, which measures complement-mediated lysis of rabbit or sheep RBCs by human serum; and the HMEC assay which measures human serum-mediated complement activation on the surface of injured human endothelial cells **(** **Figure 4** **)**.

In the Wieslab assay, the C3d mAb−CR1_1-10_ fusion proteins demonstrated nanomolar inhibitory activity in both the AP and CP terminal complex assays, consistent with the known ability of CR1_1-10_ to bind to both C3b and C4b and functionally inactivate C3/C5 convertases (8, 36) (**Figure 3**). Activity is related, at least in part, to the effector domain orientation on the targeting antibody. For example, linking CR1_1-10_ to the C-termini of either the C3d mAb heavy or light chain resulted in improved inhibitory activity compared to N-terminal fusions. This effect is particularly true of CP inhibition and suggests that accessibility of CR1_1-10_ to the convertases may be more limited when linked to the antibody N-terminus.

We also assessed the ability of the fusion proteins to inhibit complement activation using a RBC lysis assay. In this assay, complement is deposited on the RBC surface, leading to membrane attack complex (MAC)-mediated hemolysis. Results of the hemolysis assays correlated well with data from the Wieslab assays in terms of rank ordering potency of the protein constructs (**Figure 3**). The C3d mAb–CR1_1-10_ constructs demonstrated a 6- to 12-fold increase in potency in the AP assays versus soluble CR1_1-10_. This suggests that providing two moieties of CR1_1-10_ confers synergistic effector binding and inhibitory activity on AP C3/C5 convertases. This effect was not apparent in the CP assays reflecting the distinct means by which CR1_1-10_ binds to the AP convertases (via binding to C3b) versus the CP convertases (via binding to C4b/C3b).

**Figure 5:**
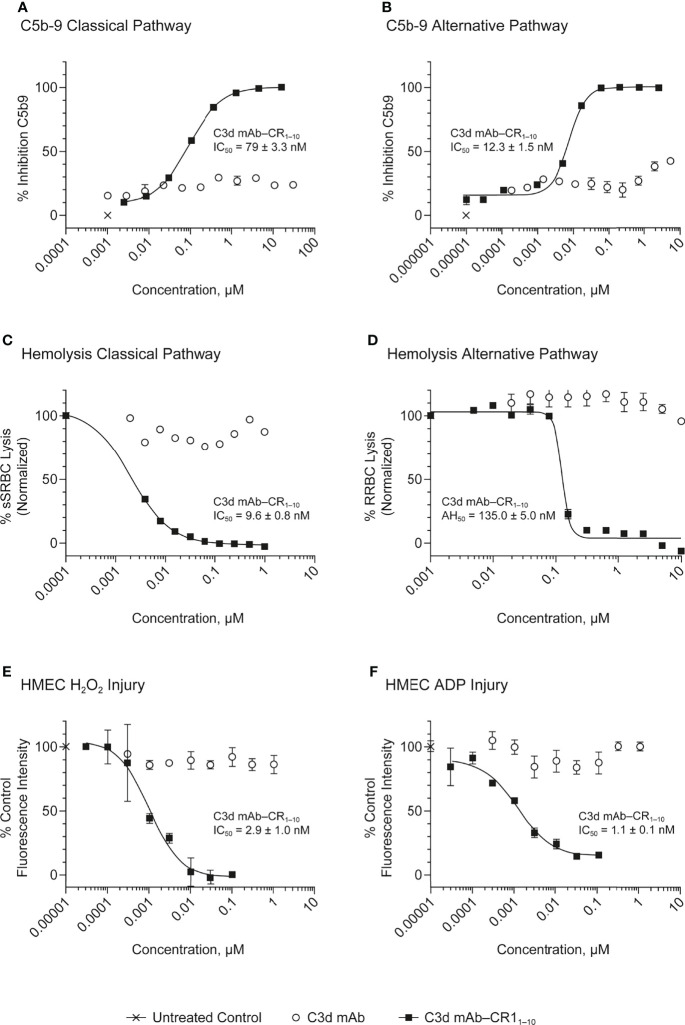
Dose response curves for C3d mAb−CR1_1-10_ and parent C3d mAb, 3d8b, in complement activation assays. **(A)** C5b-9 Classical Pathway (ELISA), **(B)** C5b-9 Alternative Pathway (ELISA), **(C)** Hemolysis Classical Pathway, **(D)** Hemolysis Alternative Pathway, **(E)** HMEC H2O2 Injury, **(F)** HMEC ADP Injury. Complement inhibitory activity is shown for C3d mAb–CR1_1-10_ (closed squares), C3d mAb (open circle) and an untreated control sample (X). The HMEC assay was carried out with either H_2_0_2_ or ADP induced injury. Replicate data are shown as mean values plus SEM. Half-maximal inhibitory concentrations in each assay are provided for C3d mAb−CR1_1-10_.

In both the Wieslab and hemolysis assays, we observed that on a molar equivalent basis, linking two CR1_1-10_ effector domains – one on each heavy or light chain – resulted in increased potency compared to soluble CR1_1-10_ or constructs containing only one CR1_1-10_ effector. This observation indicates that both effector domains can function when attached to the antibody. Furthermore, CR1_1-10_ linked to the C-terminus of either the mAb heavy or light chain consistently resulted in more potent inhibitory activity compared to fusions linked to the N-terminus. As expected, C3d mAb alone did not inhibit AP or CP complement activity in either the Wieslab **(** **Figures 5A, B** **)** or hemolysis **(** **Figures 5C, D** **)** assays.

The HMEC assay provides a direct measure of complement fragment deposition on injured human endothelial cell surfaces. In this cell-based assay, fusion proteins diluted in human serum were evaluated for their ability to inhibit C3 fragment deposition on human endothelial cells injured upon exposure to hydrogen peroxide (H_2_O_2_) or ADP. IgM and IgG present in the human serum binds to neoepitopes exposed on the injured cells, inducing CP dominant complement activation, which is measured by immunostaining with an antibody detecting C3 active fragments (37). In this system, 3d8b bound to C3d deposited on injured cells but did not inhibit deposition of C3 active fragments, confirming that 3d8b binding alone does not inhibit complement activation **(** **Figure 4** **)**. Furthermore, these data demonstrate that 3d8b binding does not interfere with C3 fragment immunostaining. In contrast, treatment of the endothelial cells with low nanomolar concentrations of C3d mAb−CR1_1-10_ fusion proteins led to potent inhibition of complement activation mediated by either H_2_O_2_ or ADP injury **(** **Figures 5E, F** **)**. We observed that the relative inhibitory activities of the fusion proteins were consistent with those of the Wieslab and hemolysis assays. We also demonstrated that in the HMEC assay, C-terminal fusion constructs outperformed the N-terminally fused constructs.

The data from these *in vitro* assays demonstrate that C3d mAb−CR1_1-10_ fusion proteins exhibit potent inhibition of AP and CP complement activity. These results also indicated that the C3d mAb containing two moieties of CR1_1-10_ linked to the C-terminus of the antibody heavy chain demonstrated the most potent inhibitory activity in all three functional assay systems. Data collected in these assays, comparing this construct to the parent antibody, confirm that the inhibitory activity of the construct is due to the negative regulator and that the antibody itself does not block complement activation **(** **Figure 5** **)**.

We further considered feasibility for manufacture and developability, including antibody titer (≥1 g/L), yield (>50%), monomer -purity (≥95%) and low propensity towards aggregation (high molecular weight ≤2%). The lead construct with CR1_1-10_ linked to the C-terminus of both heavy chains of the C3d mAb offered desirable characteristics for all of these parameters. This construct achieved a titer of 1.7 g/L by transient transfection with aggregates < 1.5%. Overall yield was 1.2g/L (66.5% recovery) following a 2-step purification process leading to 98.8% monomer with endotoxin levels <0.03 per mg. This homodimeric fusion protein has the added advantage of using a straightforward transfection and purification strategy as compared to a heterodimeric fusion protein. Given its potency and manufacture and development properties we determined that the C3d mAb fusion containing two moieties of CR1_1-10_ linked to the C-termini of both heavy chains offered the best profile for a development candidate, referred to hereafter as C3d mAb−CR1_1-10_.

### *In Vivo* Pharmacology

**Figure 6:**
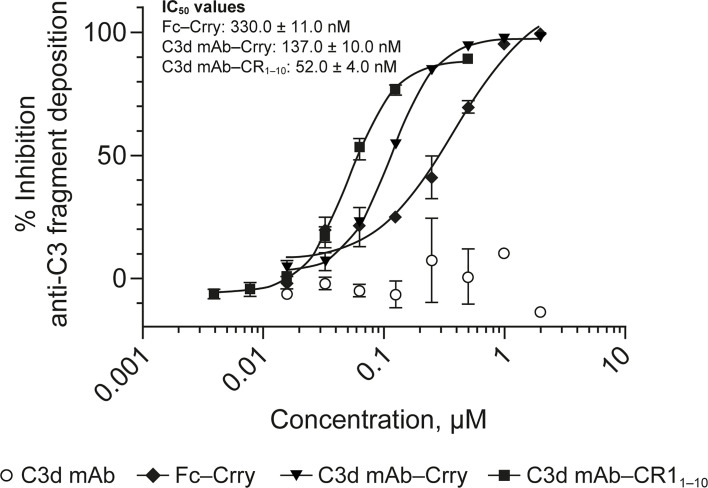
Dose response curves for mouse surrogate C3d mAb−Crry, Fc-Crry, C3d mAb-CR1_1-10_ and parent C3d mAb, 3d8b, in murine complement activation assays. Complement inhibitory activity is shown for C3d mAb–Crry (closed triangles), Fc-Crry (closed diamonds), mAb-CR1_1-10_ (closed squares) and C3d mAb (open circles) in zymosan assays using mouse serum. Replicate data are shown as mean values plus SEM. IC_50_ values are provided for C3d mAb−Crry, Fc-Crry, and C3d mAb-CR1_1-10._ mAb, monoclonal antibody; SEM, standard error of the mean.

To evaluate the advantages of C3d mAb tissue targeting of a complement negative regulatory protein, an *in vivo* proof of mechanism study was carried out using a mouse surrogate, C3d mAb−Crry. This mouse surrogate offered the advantage for limiting the development of anti-drug antibodies that could confound the interpretation of tissue PK/PD results longer than 10-days post treatment. Mouse C3d mAb–Crry demonstrated similar binding affinity to C3d as C3d mAb and C3d mAb–CR1_1-10_ (**Table 2**) and demonstrated potent inhibition of complement activation in the mouse serum zymosan assay with an IC_50_ of 140 nM (30.7 µg/mL; **Table 2**) and detectable inhibition at > 30 nM (6.5 µg/mL) (**Figure 6**). This complement inhibitory activity was similar (less than 3-fold different) to that of C3d mAb–CR1_1-10_. As a control, murine Fc−Crry was also generated and demonstrated no binding to C3d and an IC_50_ of 330 nM in the mouse zymosan assay.

#### Mouse Serum Complement Activity Assay and PK

**Figure 7:**
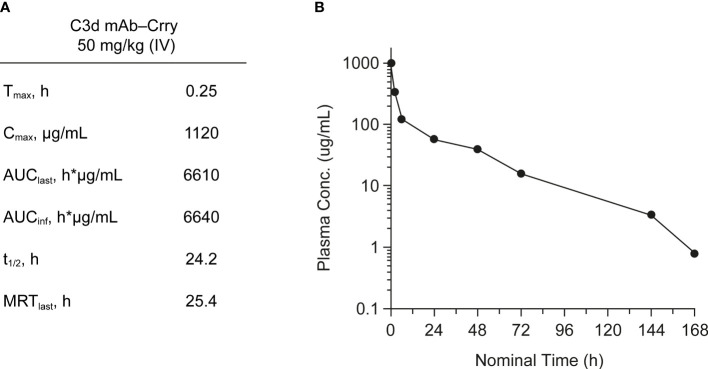
Mean pharmacokinetic parameters **(A)** and plasma concentrations **(B)** of C3d mAb−Crry post-dosing by IV administration at 50 mg/kg. AUC_last_, area under the curve from time zero to the last measurable concentration; AUC_inf_, AUC from time zero extrapolated to infinity; C_max_, maximum plasma concentration; MRT, mean residence time; t_1/2_, half-life; T_max_, time to maximum plasma concentration.

PK of C3d mAb−Crry was evaluated in wild-type C57BL/6 mice. C3d mAb−Crry demonstrated a short circulating t_1/2_ of 24.2 hours and an exposure of 6640 h*µg/mL (**Figure 7**). This was likely a result of target-mediated clearance as the C3d mAb, 3d8b, has a t_1/2_ > 9 days in C3 null mice but which is of significantly shorter duration in wild-type mice (38). Based on extrapolation of the standard curve from the mouse serum zymosan assay, this suggests that at 72 hours post-dose, the average C3d mAb−Crry circulating level of approximately 76.2 nM (16.1 µg/mL) is expected to provide approximately 25% inhibition of systemic complement activity, while at 144 hours (Day 6) the C3d mAb−Crry circulating level of 16.6 nM (3.5 µg/mL), coupled with lower drug levels at later time points is expected to provide negligible systemic complement inhibition.

#### Target Engagement

**Figure 8:**
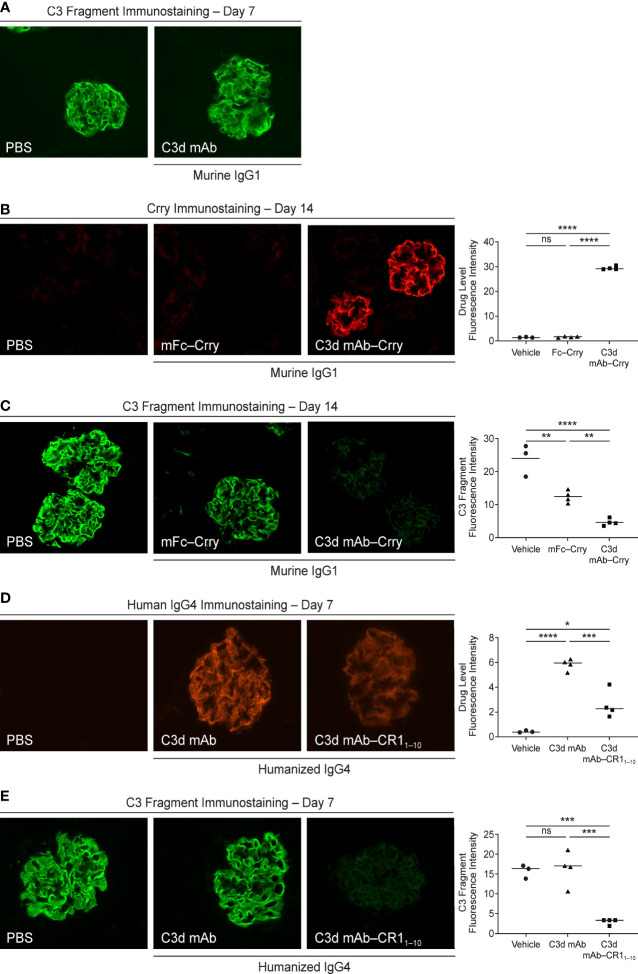
Evaluation of *CfH* ^-/-^ mouse kidney tissue for fusion protein biodistribution/tissue PK (anti-Crry or anti-hIgG4 immunostaining) and tissue PD/complement activity (anti-C3 fragment immunostaining). **(A)** C3 fragment immunostaining 7-days post-treatment with PBS and C3d mAb; **(B, C)** Crry and C3 fragment immunostaining 14-days post-treatment with PBS, C3d mAb–Crry or Fc−Crry; **(D, E)** human IgG4 and C3 fragment immunostaining 7-days post-treatment with PBS, humanized C3d mAb or humanized C3d mAb-CR1_1-10_. All images were acquired at 40x magnification. Quantitation of drug level and C3 fragment staining intensity is expressed as an average of glomerular intensity from 6 or more glomeruli per sample (ImageJ analysis) using one-way ANOVA with Tukey’s *post hoc* test. ns, not significant; *P < 0.05; **P < 0.01; ***P < 0.001; ****P < 0.0001 vs vehicle or study control.

We evaluated *in vivo* target engagement of C3d mAb-Crry in complement factor H knockout *(Cfh^-/-^)* mice. Due to the loss of functional fH protein, *CfH^-/-^* mice exhibit elevated complement activation and C3d deposition in the kidneys. This model allowed us to investigate the distribution of the fusion proteins to tissue at sites of C3d deposition and their ability to inhibit complement activity locally. As a control, *CfH^-/-^* mice were dosed with a molar matched dose of C3d mAb alone (16.9 mg/kg) and immunostained with goat anti-mouse C3 antibody to confirm no interference with the detection of complement activation occurs (**Figure 8A**). To measure target engagement and the impact on complement activity, mice were treated with 60 mg/kg C3d mAb–Crry or an equimolar amount of mFc−Crry (34.2 mg/kg) on days 1 and 2 and kidneys were harvested 14 days post-second dose. Frozen kidney sections were immunostained with a rat anti-mouse Crry antibody to detect the presence of the fusion proteins (**Figure 8B**) and with goat anti-mouse C3 antibody to monitor complement activation (**Figure 8C**). Anti-Crry immunostaining demonstrated distribution and retention of targeted fusion protein, C3d mAb−Crry, in renal glomeruli of *CfH^-/-^* mice 14-days following administration. In contrast, non-targeted fusion protein mFc−Crry did not demonstrate distribution and retention in the kidneys. Anti-C3 immunostaining of kidney sections from C3d mAb–Crry treated mice also demonstrated near complete inhibition of complement activation in the glomeruli 14 days after dosing, while mFc−Crry treatment showed partial inhibition of complement activation and likely through systemic inhibition of complement activity. These data demonstrate that the C3d mAb fusion protein effectively distributes to kidney where C3d is deposited and provides long-term durable tissue PK and sustained inhibition of complement activation. Having demonstrated the intended mechanism of action, we further evaluated the *in vivo* activity of our lead fusion protein, humanized C3d mAb – CR1_1-10_. A single dose of humanized C3d mAb-CR1_1-10_ (10 mg/kg), humanized C3d mAb (5 mg/kg) and PBS were administered on day 1 and kidneys were harvested at day 8. Kidney sections were immunostained with an anti-human IgG4 antibody to evaluate target engagement and with an anti-C3 antibody to monitor complement activation (**Figure 8D, E**). C3d mAb and C3d mAb-CR1_1-10_ demonstrated the ability to distribute into kidney tissue and maintain detectable target engagement 7 days post-dosing. C3d mAb-CR1_1-10_ treatment results in near complete inhibition of complement activation in the kidney tissue while the C3d mAb treatment maintains similar levels of complement activity as the untreated PBS control.

## Discussion

We describe the generation of antibody-derived C3d-targeted fusion proteins that are designed to deliver a fragment of the complement negative regulatory protein, CR1, to tissues where complement is over-active. This provides a means to effectively inhibit complement in the diseased tissue without long-term systemic blockade. When complement is activated, C3b is first formed by C3 convertases and serves as a component of both the amplification loop and the C5 convertase. To regulate this process, C3b is inactivated by factor I and cofactor-driven cleavage, first to iC3b and then C3dg, neither of which can support further complement activation. C3dg is further cleaved by a protease to form the C3d fragment. These C3 cleavage products are deposited at high density in tissue through covalent binding *via* an exposed common thioester domain (TED) (38) at sites of complement activation (27–30).

To develop a tissue-localized complement inhibitor, we selected C3d as the tissue target. Previous studies have identified a mouse anti-C3d antibody, 3d8b, that binds to a common epitope shared by iC3b, C3dg, and C3d with low nanomolar affinity, but does not bind to C3 or C3b (26). This antibody was used as the anti-C3d targeting domain of our fusion protein constructs. A combination of peptide mapping and co-crystallization studies were designed to characterize the binding interactions of 3d8b with C3d. We identified the epitope, which is located at the C-terminus of C3d, a site common to the TED containing C3 fragments iC3b, C3dg and C3d. We also demonstrated that the binding affinity of this antibody to deposited C3d is in the low nanomolar range. Thus, a C3d mAb fusion protein that binds with high affinity to covalently linked C3d provides the potential for durable tissue PK and PD properties. In previously described studies of the fusion constructs TT30 and TT32, which were also designed as tissue targeted complement inhibitors, CR2 was used as a C3d targeting domain; however, our epitope mapping studies indicate 3d8b binds to a C3d epitope distinct from that of CR2 (39). In addition, 3d8b binds to deposited C3d with 200- to 1000-fold better affinity, as compared to CR2 (unpublished data).

The first 10 consensus repeats of CR1 (CR1_1-10_) have been shown to potently inhibit all C3/C5 convertases and thus all three pathways of the complement system (AP, CP and LP) (40–42). Our fusion constructs were designed so that when the antibody portion binds to C3d in diseased tissue it brings the human CR1_1-10_ protein into close proximity with surface-bound C3/C5 AP convertases, allowing CR1_1-10_ to interrupt ongoing and further complement activation. To test this concept a series of C3d mAb–CR1_1-10_ recombinant fusion proteins were engineered and expressed in mammalian cells and evaluated for their binding to C3d and ability to inhibit AP and CP complement activation in biochemical and cell-based functional assays. These studies allowed us to identify the scaffolds that retain high-affinity C3d binding and potent *in vitro* complement inhibitory activity. They also allowed us to demonstrate that engineering protein constructs that contained two moieties of CR1_1-10_ linked to the C-terminus of an anti-C3d mAb Fc domain provided synergistic potency in multiple *in vitro* complement assays versus molar matched concentrations of soluble CR1_1-10_ protein.

We evaluated *in vivo* biodistribution and tissue PK and PD of a lead prototype fusion protein containing two moieties of the murine orthologue of human CR1, Crry, linked to the C-terminal end of the Fc domain of a C3d mAb (C3d mAb–Crry) in *CfH^-/-^* mice. These mice lack expression of the fH protein and have uncontrolled complement AP activation leading to excessive C3 fragment deposition in their kidneys. We demonstrated that this prototype fusion protein, C3d mAb–Crry, distributed to the kidneys of *CfH^-/-^* mice where C3d is deposited and showed durable tissue PK and PD (inhibition of complement activation) for 14 days post-dosing. Conversely, administration of a molar-matched concentration of a non-targeting Fc-Crry construct demonstrated no tissue residency and minor inhibition of complement activity in the kidneys of *CfH^-/-^* mice at day 14. PK studies demonstrated that circulating levels of C3d mAb–Crry administered at the same dose in the *CfH^-/-^* mice are effectively below the levels associated with systemic complement inhibition 7 days post-dosing. These studies effectively demonstrate that the inhibition of complement detected in target organs are a result of tissue targeting. They also demonstrate that C3d mAb–Crry exhibits a short circulating exposure and long-term durable tissue PK. The short circulating exposure is expected because of target-mediated clearance of C3d mAb binding to C3d epitope that is also exposed in iC3b and potentially C3 (H_2_O) produced during C3 complement tick over. This is consistent with data that demonstrate 3d8b has a half-life in C3-deficient mice of > 9 days and considerably shorter in wild-type mice (38). Follow on studies administering a single, 10 mg/kg dose of the humanized C3d mAb and humanized C3d mAb-CR1_1-10_ recapitulate the tissue targeting and effective inhibition of complement activation as demonstrated by the mouse surrogate. These data validate the potential for further therapeutic development of a C3d tissue targeted complement negative regulatory protein.

While the complement system represents an indispensable arm of the innate immune system, it also has the potential, through uncontrolled activation, to act as an initiator or to play a significant role in numerous autoimmune and inflammatory disorders. Inhibition of complement therefore represents a potential therapeutic strategy for many diseases. Two recently approved anti-complement therapies that block terminal complement C5 activation provide validation of complement as a therapeutic target but also reveal inherent limitations in systemic inhibition as a therapeutic approach. Soliris (eculizumab) received US Food and Drug Administration (FDA) approval in 2007 for PNH and was subsequently approved for several additional indications. A version with extended half-life, Ultomiris (ravulizumab-cwvz), received US FDA approval for PNH in 2018. However, systemic complement blockade is associated with substantially increased risks of bacterial infections, due to complement’s role in controlling the innate immune response to pathogens. Patients treated with eculizumab have approximately a 1,000- to 2,000-fold increased risk of meningococcal infection/sepsis (*Neisseria meningitidis*), with a reported incidence of up to 1.5% (23–25). This increased risk is further complicated by the fact that standard-of-care treatment for most complement-mediated diseases outlined above includes the use of immunosuppressive agents, each carrying individual and aggregate safety concerns(43). For this reason, eculizumab and ravulizumab include special warnings in their product labelling and corresponding measures in their risk management plans (RMP) and risk evaluation and mitigation strategy (REMS), including receipt of the meningococcal vaccine in advance of treatment. Additionally, the high abundance and rapid systemic turnover of complement components requires sustained, high pharmacologic concentrations of systemic complement inhibitors to overcome this endogenous circulating pharmacologic ‘sink’ to be effective. Overcoming these challenges with a therapeutic agent requires a difficult-to-attain balance between circulating concentrations that are sufficient to effectively inhibit tissue complement but low enough to minimize infection risk to patients. An unmet need remains, therefore, for safer and more efficacious anti-complement therapies, particularly among patients who concurrently receive other immunosuppressive therapies. Targeted tissue delivery of a complement inhibitor may ameliorate this treatment gap, by enabling longer lasting inhibition at the sites of injury, with derivative safety and efficacy benefits.

To address this unmet need, we have adopted a more targeted therapeutic approach whereby complement regulation is restored specifically in diseased tissue, avoiding the potential consequences of systemic blockade, and providing potential efficacy and safety benefits. This approach focused on developing bifunctional fusion proteins that include an antibody that binds with high affinity to C3d, which is deposited in diseased or injured tissues at sites of complement activation (27–30). Upon binding to C3d, the fusion protein presents CR1_1-10_ for binding to the C3b and C4b components of the C3/C5 convertases, potently inactivating all the complement pathway convertases by dissociation (decay accelerating activity) and irreversible catalytic degradation (co-factor activity). Thus, the C3d mAb−CR1_1-10_ fusion protein has the potential to restore proper complement regulation through a unique, tissue-targeted therapeutic approach and can effectively inhibit all three pathways of the complement system: CP, LP and AP. In addition, C3d has a slow turnover time in diseased tissue in the order of days to weeks, suggesting that a C3d mAb fusion protein has the potential for long-term, durable tissue PD (complement inhibition).

We have shown that a fusion protein combining an anti-C3d mAb linked to CR1_1–10_, maintains high affinity binding to C3d and exhibits potent complement inhibitory activity in several *in vitro* assay systems. Furthermore, *in vivo* studies with a mouse surrogate, C3d mAb-Crry, demonstrate distribution to diseased kidneys at sites where C3d is deposited and potent and durable tissue PK and PD, in the presence of short circulating PK. These characteristics suggest that tissue targeted complement negative regulators can achieve robust efficacy while minimizing circulating drug exposure that may affect immune surveillance or other homeostatic functions mediated by systemic complement activity. Given the hallmark of C3d deposition in injured and diseased tissue where complement is activated, anti-C3d mAb fusion proteins have therapeutic potential for multiple clinical indications. Finally, given the ability of complement regulatory proteins to elaborate differential activity against the complement pathways and activation steps, expansion of this targeting concept will include other inhibitory molecules to modulate complement activity.

## Data Availability Statement

The datasets presented in this study can be found in online repositories. The name of the repository and accession number can be found below: Research Collaboratory for Structural Bioinformatics (RCSB) Protein Data Bank (PDB), https://www.rcsb.org/, 7UE9.

## Ethics Statement

The animal study was reviewed and approved by Biomere’s Institutional Animal Care and Use Committee (IACUC).

## Author Contributions

SV, SK, FL, KF, VH, JT, MS, SR were responsible for the conception and design of the study, while SV, MS, FT, ES and KF were responsible for the conception and design of the fusion protein reagents used in the study. FL, JM, SR, KF performed the *in vitro* assays and analyzed the data, while FL and SR performed and analyzed the immunostaining assays. SK, SV, FL and SR reviewed and interpreted immunostaining and PK data. KF and SV were responsible for drafting the manuscript, while all authors revised the manuscript critically for intellectual content, approved the final version to be published, and agreed to be accountable for all aspects of the work.

## Conflict of Interest

KF, FL, JM, SR, SW, and SV are employees and shareholders of Q32 Bio Inc. SK and MS were previous employees of Q32 Bio Inc. and are shareholders of Q32 Bio Inc. SK is currently an employee of Compass Therapeutics. MS is currently an employee of Sanofi. ES is a paid consultant of Q32 Bio Inc. FT was previously a paid consultant of Q32 Bio Inc. JT is an employee of the University of Colorado School of Medicine and a consultant, founder, and shareholder of Q32 Bio Inc. VH is an employee of the University of Colorado School of Medicine; a founder, advisor, shareholder and previous consultant of Q32 Bio Inc. KF, FL, JM, SR, SW, SV, SK, MS, VH, and JT acknowledge a potential conflict of interest related to their relationship with Q32 Bio Inc.

This work was funded by Q32 Bio Inc. (Waltham, MA, United States). The funder had the following involvement: study design, data collection, analysis and interpretation of data, preparation of this manuscript and the decision to submit it for publication. Medical writing and editorial support provided by Paul Littlebury, PhD, of Bioscript Stirling (Macclesfield, UK), was funded by Q32 Bio Inc.

## Publisher’s Note

All claims expressed in this article are solely those of the authors and do not necessarily represent those of their affiliated organizations, or those of the publisher, the editors and the reviewers. Any product that may be evaluated in this article, or claim that may be made by its manufacturer, is not guaranteed or endorsed by the publisher.

## Supplementary Material

Supplementary Figure 1 Purity of C3d mAb–CR11-10 fusion and control proteins by Size-Exclusion – High Performance Liquid Chromatography (SEC-HPLC) and Sodium Dodecyl-Sulfate Polyacrylamide Gel Electrophoresis (SDS-PAGE). Proteins were analyzed by SEC-HPLC on a TSKgel G3000SWXL or G4000SWXL, depending on molecular weight of the protein undergoing analysis, with PBS, pH 7.4 mobile phase for separation. 5 µg of each protein after buffer exchange and concentration was separated under reducing and non-reducing conditions in 2-[N-morpholino]ethanesulfonic acid (MES) Running buffer on 4–12% Bis-Tris pre-cast NuPAGE gels.

Supplementary Figure 2 Binding affinity of anti-C3d mAb and lead mAb-fusion protein to C3d. Binding affinity to C3d is unaffected by inclusion of CR11-10 (~2-fold) or Crry (no change) fusion proteins.
