# Supplementary figures and images for: Development and Optimization of Bifunctional Fusion Proteins to Locally Modulate Complement Activation in Diseased Tissue
Source: Front Immunol. 2022 Jun 16;13:869725. doi: 10.3389/fimmu.2022.869725 (PMC9244803; doi:10.3389/fimmu.2022.869725)

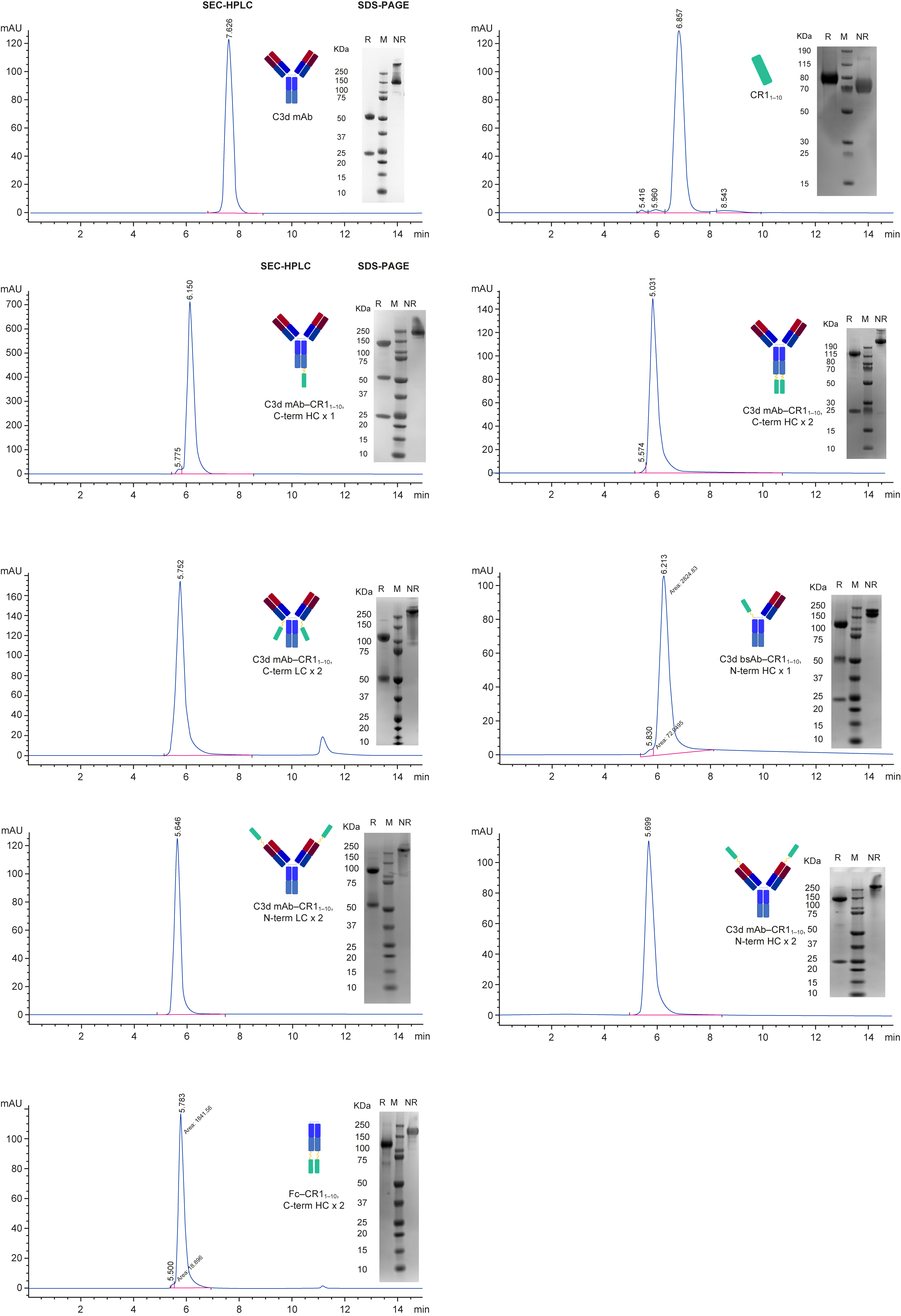

Supplement: Supplementary Figure 1 — Purity of C3d mAb–CR11-10 fusion and control proteins by Size-Exclusion – High Performance Liquid Chromatography (SEC-HPLC) and Sodium Dodecyl-Sulfate Polyacrylamide Gel Electrophoresis (SDS-PAGE). Proteins were analyzed by SEC-HPLC on a TSKgel G3000SWXL or G4000SWXL, depending on molecular weight of the protein undergoing analysis, with PBS, pH 7.4 mobile phase for separation. 5 µg of each protein after buffer exchange and concentration was separated under reducing and non-reducing conditions in 2-[N-morpholino]ethanesulfonic acid (MES) Running buffer on 4–12% Bis-Tris pre-cast NuPAGE gels. [file Image_1.tif]

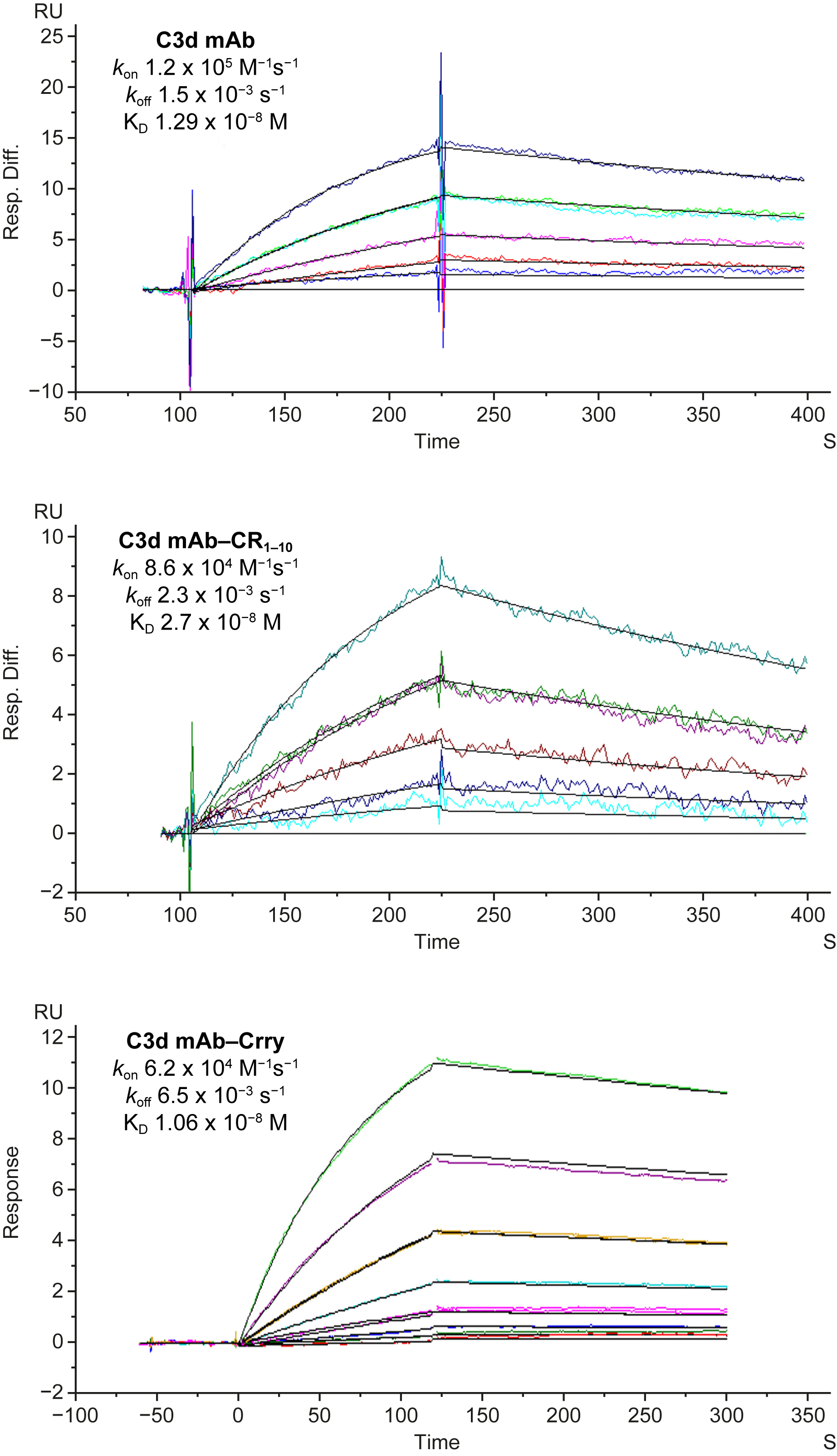

Supplement: Supplementary Figure 2 — Binding affinity of anti-C3d mAb and lead mAb-fusion protein to C3d. Binding affinity to C3d is unaffected by inclusion of CR11-10 (~2-fold) or Crry (no change) fusion proteins. [file Image_2.tif]
